# Practical Preventive Considerations for Reducing the Public Health Burden of Poultry-Related Salmonellosis

**DOI:** 10.3390/ijerph20176654

**Published:** 2023-08-25

**Authors:** Rabin Raut, Pramir Maharjan, Aliyar Cyrus Fouladkhah

**Affiliations:** 1 Cooperative Extension Program, Tennessee State University, Nashville, TN 37209, USA; rraut@tnstate.edu; 2 Public Health Microbiology Laboratory, Tennessee State University, Nashville, TN 37209, USA; 3 Public Health Microbiology FoundationSM, Nashville, TN 37209, USA

**Keywords:** *Salmonella* serovars, poultry, antimicrobial resistance, food chain

## Abstract

With poultry products as one of the leading reservoirs for the pathogen, in a typical year in the United States, it is estimated that over one million individuals contract non-typhoidal *Salmonella* infections. Foodborne outbreaks associated with *Salmonella* infections in poultry, thus, continue to remain a significant risk to public health. Moreover, the further emergence of antimicrobial resistance among various serovars of *Salmonella* is an additional public health concern. Feeding-based strategies (such as use of prebiotics, probiotics, and/or phytobiotics as well as essential oils), non-feeding-based strategies (such as use of bacteriophages, vaccinations, and in ovo strategies), omics tools and surveillance for identifying antibiotic-resistance genes, post-harvest application of antimicrobials, and biosecurity measures at poultry facilities are practical interventions that could reduce the public health burden of salmonellosis and antibiotic resistance associated with poultry products. With the escalating consumption of poultry products around the globe, the fate, prevalence, and transmission of *Salmonella* in agricultural settings and various poultry-processing facilities are major public health challenges demanding integrated control measures throughout the food chain. Implementation of practical preventive measures discussed in the current study could appreciably reduce the public health burden of foodborne salmonellosis associated with poultry products.

## 1. Introduction 

The *Salmonella* serovars have a complex and evolving nomenclature and since their discovery over 100 years ago, they continue to be a major global, national, and regional public health challenge [1,2,3]. In a typical year, more than one million individuals are estimated to contract non-typhoidal *Salmonella* infections in the United States [4]. Similarly, around 27 million global cases of salmonellosis are estimated to be associated with typhoidal *Salmonella* serovars [5]. Various typhoidal and non-typhoidal *Salmonella* serovars are capable of forming complex biofilms on biotic and abiotic surfaces, further complicating the challenges associated with the control of this prevalent and opportunistic pathogen [6,7,8]. Under the landscape of a changing climate, the public health challenges associated with *Salmonella* serovars, and with antibiotic resistance are expected to be further augmented in the future [9,10]. Poultry-processing facilities and poultry products are among the main reservoirs of both typhoidal and non-typhoidal serovars of *Salmonella*.

Poultry contributes significantly to meat and egg production around the globe. According to the global livestock environmental assessment (GLEAM) conducted by the Food and Agriculture Organization (FAO), the production of eggs in 2016 was estimated at 73 million tons, while the production of chicken meat was estimated at 100 million tons. These statistics are expected to increase in the coming years because of the increases in global population, higher incomes, and rapid urbanization [11,12,13]. The increasing consumption of meat, mainly chicken and turkey, is due to the lower cost incurred in production [14]. Poultry, as a major source of animal protein, is typically considered superior to plant-based protein in providing micronutrients such as vitamin A, vitamin B, iron, zinc, and calcium in addition to all essential amino acids [11]. Moreover, it has no major restrictions for consumption in communities holding different religious beliefs [11,15].

In the last seventy years, not a single poultry-producing area of the world has been free of foodborne bacteria [16]. Meat and eggs can be contaminated and re-contaminated with different species of bacteria. *Salmonella* serovars, *Campylobacter jejuni*, *Campylobacter coli*, and *Clostridium perfringens* are more frequently detected as the major pathogens of meat and eggs [17]. Besides these, *Staphylococcus aureus*, *Listeria monocytogenes*, and pathogenic serogroups of *Escherichia coli* are abundant in various value-added poultry products [16,17]. Among the plethora of bacteria associated with poultry, *Salmonella* serovars have been the most important pathogen of public health concern in poultry meat [18,19].

Despite the robust control and preventive approaches applied against *Salmonella*, foodborne outbreaks associated with *Salmonella* are still a serious concern [20,21]. The age and genetics of the birds, the stress induced by overstocking, the extent of pathogen exposure, and the virulence of the specific strain contribute to the colonization of *Salmonella* in broilers [22,23]. *Salmonella* can infect broilers immediately after hatching and for the first few days by vertical or horizontal transmissions in hatcheries during activities such as feeding, handling, and transportation [24,25]. Moreover, ingestion of contaminated litter, and changes in crop pH and intestinal microbiota predispose the birds to higher susceptibility [26,27].

*Salmonella* is a Gram-negative bacterium that primarily inhabits the gastrointestinal tract of many warm-blooded animals. It is a facultative anaerobe, meaning it can survive with or without oxygen, and it does not form spores [28]. Most *Salmonella* serovars possess peritrichous flagella, allowing them to exhibit motility and move in various directions. However, there are notable exemptions including *Salmonella* Pullorum and *Salmonella* Gallinarum which are typically considered to be non-motile but are highly pathogenic to poultry [29]. As a non-fastidious bacterium, *Salmonella* can colonize and replicate even under adverse environmental conditions on various biotic and abiotic surfaces. It is capable of multiplication at water activity levels as low as 0.94 and can withstand a pH range of 3.7 to 9.5 [30]. The prevalence of *Salmonella* is common in dairy products, meat products, and fresh produce contaminated with poultry-industry by-products [31]. The different forms of salmonellosis in humans are typhoid, paratyphoid, and non-typhoid *Salmonella* infections [4,32,33].

Poultry products are linked to 29% of *Salmonella* infections [34,35,36,37]. Salmonellosis in poultry is recognized as a zoonotic disease with economic and public health implications on a global scale [20,38,39,40]. Poultry meat and various value-added poultry products are considered as two of the main reservoirs of various *Salmonella* serovars with *Salmonella* Enteritidis and *Salmonella* Typhimurium being among the most epidemiologically significant serovars associated with poultry-related outbreaks.

**Figure 1:**
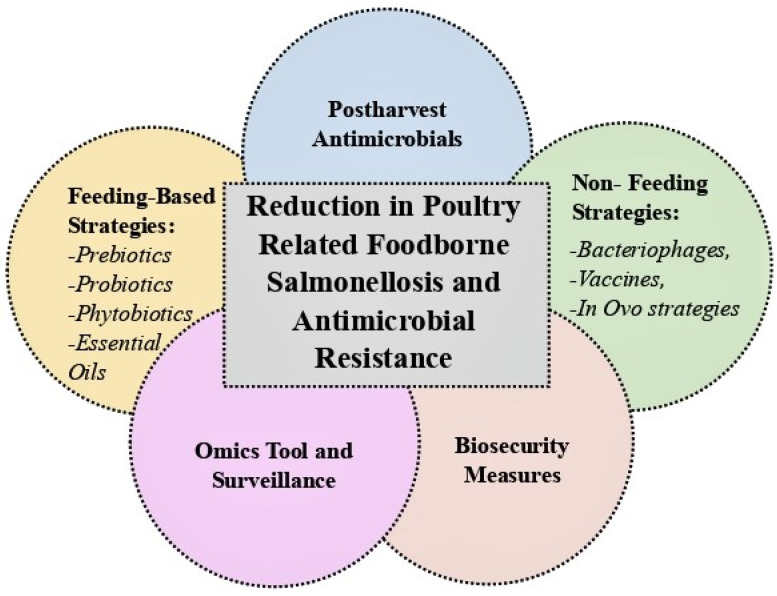
Practical considerations for reducing the public health burden of foodborne salmonellosis associated with poultry products.

Some specific serovars of *Salmonella* can exhibit a high degree of antimicrobial resistance, and thus they have drawn the attention of stakeholders and policymakers. The spread of *S. enterica* serovar Typhimurium DT104, as an example, along with other multi-drug-resistant strains are partly responsible for the spread of antibiotic-resistant *Salmonella* in the poultry food chain [41]. Additionally, some new strains of *S. Kentucky* continue to illustrate resistance toward carbapenems and fluoroquinolones, and may bring life-threatening diseases in humans. These emerging and re-emerging *Salmonella* serovars with antibiotic-resistance characteristics highlight the public health importance of infectious diseases associated with poultry products [42]. The rampant use of antimicrobials in both animal production and the treatment of human and animal diseases could be a contributing factor in *Salmonella* serovars’ resistance to one or more antibiotics [43]. And poultry is more prone to continuous use of antibiotics as it alters the gut microbiota which may have negative effects on overall health and thus the optimal production of meat or eggs. As such, antibiotic-free (ABF) strategies promote alternative methods for disease prevention, improved animal health, and the production of safer poultry products, thus contributing to the overall sustainability and health of the poultry industry. Different alternatives, like herbal plants, organic acids, prebiotics, and probiotics, are available for the purpose of growth promotion. Better on-farm management strategies such as the availability of clean water, and robust biosecurity measures can aid in reducing the public health burden of *Salmonella* [44]. In the ABF approach, feeding-based and non-feeding-based interventions are in existence to control the *Salmonella* burden in poultry flocks. Strategies involving the modification of poultry feed (such as use of prebiotics, probiotics, synbiotics, postbiotics, and phytobiotics) involve using substances and/or microorganisms that are consumed to promote healthy gut microbiota. On the other hand, non-feeding-based strategies, such as vaccinations, application of bacteriophages, and in ovo interventions are methods that do not involve direct consumption and aim to modulate the gut microbiota or target specific pathogens [28]. The purpose of the current study was to examine the fate, prevalence, and transmission of *Salmonella* in poultry-processing facilities and discuss practical interventions to reduce the public health burden of poultry-related Salmonellosis (Figure 1).

## 2. Identification of *Salmonella* Serovars

Although *Salmonella* has a complex nomenclature, the bacterium genus consists of only two species—*S. enterica* and *S. bongri*. While the latter is not typically associated with human health complications, the subspecies of *S. enterica* are enterica, arizonae salamae, indica, diarizonae, and houtenae which could be pathogenic to humans. Isolates from these species and subspecies are frequently classified according to their antigenic characteristics and over 2600 serovars are reported to exist [45]. The division of *Salmonella* serovars is done as per the O (somatic), H (flagellar), and Vi (antigen) combinations. The O antigens are present on the surface of the outer membrane, the H antigens are found in the flagella, and the Vi antigens overlie the O antigens [46]. Identification based on this method is used to serotype more than 2600 serovars currently in existence [47]. With the rapid rise of serovars, identification was simplified to three types of *Salmonella*, namely, non-typhoidal *Salmonella*, *Salmonella* typhi, and *Salmonella* paratyphi [48]. Non-typhoidal *Salmonella* infections cause dehydration, elevated body temperature, episodes of vomiting, and discomfort in the abdominal region in humans, typically 6–72 h after exposure [49]. Patients suffering from typhoidal salmonellosis typically have symptoms such as headache, body aches, fever, constipation, or diarrhea [48]. The infective doses of different *Salmonella* serotypes can vary depending on the type of food involved and the susceptibility of the patient. In the case of fat-rich foods, such as certain dairy products, the bacterium has been found to exhibit enhanced survival. As a result, even a relatively small number of the bacterial cells, usually fewer than 100 cells, could be sufficient to cause illness if ingested [50]. Therefore, implementing a higher standard of detection in various products including ready-to-eat (RTE) foods would be critical to safeguard the public health [51].

## 3. Colonization of *Salmonella* Serovars in Poultry Flocks

When litter is contaminated with a high dose of *Salmonella* serovars, the bacterium could understandably colonize in the intestine of poultry and, due to genetic similarity of the birds, the bacterium could subsequently spread rapidly in the flock [52]. Then, it competes with other microflora occupying its niche that supply nutrients for its multiplication, and invades the host’s immune system [53]. *Salmonella* is well-equipped with virulent factors, prophages, and plasmids (mobile genetic elements) that enable it to cause infections in the host’s gut [54,55]. *Salmonella* pathogenic island 1 (SPI-1), a genetic cluster, is responsible for invading the enterocyte by generating a type III secretion system, a mechanism used by the bacterium to secrete effector proteins into the cells of the host. Similarly, other SPIs (such as SPIs 2 to 4), enable survival of the bacterium in biotic environments and subsequently lead to the release of secondary metabolites during infections in gastrointestinal areas. The bacterium then needs other pathogenic islands to adapt to other specific environments and hosts [56,57]. In the 20th century, outbreaks associated with *Salmonella* Gallinarum were worrisome on commercial farms globally [58]. Following that period, public health was threatened by the emergence of *S. Typhimurium* and *S. Enteritidis* in poultry flocks and food items such as chicken and turkey meat and eggs [59,60,61]. During the 1990s, *S. Enteritidis* outnumbered other serovars in poultry environments, and foodborne illness associated with it was noticed in many countries [62]. In recent years, *Salmonella* serovars like Heidelberg, Minnesota, Montevideo, Tennessee, and Kentucky continued to rise within poultry food chains and food products at a global scale [8,63,64]. The use of phages as an alternative to antibiotics has other co-benefits as well. As an example, phages, due to their specificity to pathogens, typically do not interfere with the commensal microflora of poultry [65] and could be used in combination with prebiotic and/or probiotic additives.

## 4. *Salmonella* in the Poultry Production System at the Preharvest Level

Poultry can acquire *Salmonella* infections from different sources such as contaminated feed, breeder flocks, hatcheries, farm environments, contaminated litter, and feed withdrawal.

### 4.1. Feed

Various *Salmonella* serovars are able to survive in a wide range of environmental conditions and, additionally, can survive low-moisture conditions for extended periods of time, making this pathogen an important pathogen of concern in animal feed. Application of thermal processing of the feed could eliminate the pathogen but *Salmonella* could be re-introduced to the feed in poultry-processing facilities [66].

Feed, as a potential source of *Salmonella*, can compromise the bird’s performance if contaminated with pathogens of poultry health concern. The infected birds that consume contaminated feed excrete *Salmonella* into the farm environment. Non-uniform distribution of microorganisms in the feed could complicate the proper detection of *Salmonella* in poultry feed. Additionally, sub-lethally injured but viable *Salmonella* might not be detected properly using traditional laboratory analysis [37]. Raw feed ingredients can be contaminated by *Salmonella* at several stages of manufacturing. The multiplication of microorganisms at the feed plant is affected by moisture, the composition of the feed, and the thermal processing intensity applied during preparation.

### 4.2. Parent Stock and Hatchery

Broiler breeders require vaccination to control the vertical transmission of *Salmonella* serovars to the hatching progeny and minimize their distribution in the processing plant [67]. A study of vaccinated breeders showed a significantly lower *Salmonella* load in the ceca (38%) and reproductive tracts (14.22%) than in non-vaccinated breeders (64.2% and 51.7%). Consequently, the progeny of vaccinated breeders also exhibited a lower population of *Salmonella* (18.1%) when compared to non-vaccinated breeders (33.5%) [68]. Infection of a hatching chick at the hatchery is through vertical and/or horizontal transmission. Vertical transmission is due to the laying of eggs by contaminated poultry [69,70]. In horizontal transmission, newly hatched chicks are exposed to *Salmonella* through the hatching of contaminated eggs. Furthermore, exposure to contaminants at the farm, could contaminate the outer surface of the eggshell and could penetrate into the egg [71]. Epidemiological studies further indicate that horizontal transmission is an important aspect due to the genetic similarities of the birds and the prevalence of various *Salmonella* serovars in poultry production facilities [72]. 

### 4.3. Litter

Litter offers comfort to broiler chickens as a source of bedding material [73]. Good litter management contributes to poultry welfare, disease prevention, and more efficiency in production. Litter is a significant source of dust [74] and thus could be a source for *Salmonella* survival and multiplication. Dust-related transmission is mainly remediated by controlling the moisture content of the litter [75]. Various studies have identified the relationship between strains detected in litter and broiler carcass contamination. Thus, the *Salmonella* prevalence in the litter at the rearing facilities can disseminate to poultry carcasses during processing [76].

### 4.4. Feed Withdrawal

Before transferring broilers to the processing mill, withdrawing feed for 8 to 12 h is a common practice in some facilities. However, feed withdrawal has been found to be a cause of the increased presence of *Salmonella* in the ceca and poultry products [77]. In search of food under feed-deprived environments, birds may be exposed to contaminated litter and this may lead to contamination of the ceca and poultry products. Some studies have shown the association of *Salmonella* prevalence in poultry products with a drop in lactic acid production. Feed withdrawal increases pH in the crop as a result of a drop in *Lactobacillus* fermentation [73].

## 5. Contribution of Poultry as a Reservoir of Antimicrobial Resistance

Poultry products are one of the leading contributing factors to *Salmonella* infections in humans [78]. Additionally, the excessive therapeutic and sub-therapeutic application of various antibiotics in the poultry food chain in various regions of the world is a potential contributing factor to the prevalence of antimicrobial-resistant bacteria in the food chain [79]. The increasing resistance of different *Salmonella* isolates to various antibiotics that are intended to treat invasive infections is a matter of great concern for public health [5]. As the efficacy of traditional antibiotics such as ampicillin, chloramphenicol, and trimethoprim–sulfamethoxazole has reduced in recent years, fluoroquinolones and broad-spectrum cephalosporins have been the drugs of choice to treat salmonellosis [80,81]. The link between human infections and non-typhoidal *Salmonella* and poultry products has been well understood [78,82]. Modern intensive poultry production in many regions of the world utilizes antibiotics, both for growth (sub-therapeutic) and treatment (therapeutic) purposes. Zoonotic disease can be transmitted to humans through three different routes: (i) direct contact between humans and animals; (ii) consumption of contaminated food; and (iii) exposure to a contaminated environment [83]. Eggs and meat can be contaminated with water and feed containing antibiotic-resistant bacteria (ARB) harboring antibiotic-resistant genes (ARGs). This makes the food chain one of the significant routes of ARB transmission [84]. For instance, the excessive use of enrofloxacin in poultry farming, in the 1990s is potentially responsible for the reduced susceptibility of *S. Typhimurium* DT104 to ciprofloxacin isolated from poultry food and subsequently from human isolates. The connection between the use of nitrofurans in poultry-producing regions and the persistence of *S. Enteritidis* in poultry has been similarly established. This correlation has led to an increased risk of human salmonellosis in Portugal, as an example, due to the transmission of antibiotic-resistant *Salmonella* through the food chain [85]. Another observational study of poultry producers in Canada also points to the potential linkage between the poultry industry and the prevalence of ceftiofur-resistant *S. Heidelberg*, a significant serotype of *Salmonella* in poultry production. The prevalence of *S. Heidelberg* was reduced due to the voluntary withdrawal of ceftiofur from both human infection treatments and retail poultry, however, resistance levels again rose after the reintroduction of antibiotic use [86].

A review of recent observational studies indicates that poultry and poultry products are an important reservoir for antibiotic-resistant bacteria in food chains, with a high tendency for dissemination to humans [87]. In addition to *Salmonella* serovars, Gram-positive bacteria are considered as a main reservoir for class 1 antibiotic resistance integrons in poultry production facilities as well [88]. In harmony with the overall recommended approach of the current study, other studies also emphasize the critical importance of prevention as well as incorporating a holistic “one health” approach for reducing the public health burden of antibiotic-resistant microorganisms associated with poultry products and production [89].

## 6. Control and Prevention of *Salmonella*

Controlling *Salmonella* in poultry productions and poultry meat, and thus preventing its entry into the food chain requires multiple interventions. As discussed earlier, the threat of ARB increases by administering antibiotics in the poultry industry for prophylactic and therapeutic purposes. Thus, non-antibiotic alternatives in poultry production have gained increasing attention in recent years. These alternatives are feeding-based interventions, such as probiotics, prebiotics, phytobiotics, and postbiotics. In addition to the feeding-based approach, non-feeding-based alternatives focused on using bacteriophages, vaccines, and in ovo strategies are also common practical interventions in the poultry industry to minimize the *Salmonella* burden [28]. More recently, whole genome sequencing (WGS), an application of genomics, has been used in the diagnosis, epidemiological studies, and surveillance of *Salmonella* [90].

### 6.1. Feeding-Based Strategies

#### 6.1.1. Prebiotics

Probiotics are types of food ingredients that have a positive impact on the host’s health by selectively promoting the multiplication and activity of specific bacteria in the colon. This definition emphasizes the non-digestible nature of prebiotics and their ability to improve the overall well-being of the host through the modulation of beneficial gut bacteria. [91]. Improvement in broiler growth is achieved through the increased production of amylase within the intestinal tract of chickens [92] and the minimization of *Salmonella* colonization during hen molting [93]. Some studies have indicated the potential of prebiotics to combat *Salmonella* by providing binding sites for bacteria to be excreted out of the digestive tract [94]. Some studies have reported a drop in *Salmonella* numbers by supplementing prebiotics, which increases the short-chain fatty acid (SCFA) concentrations [95]. Prebiotics like trehalose and *Aspergillus* meal prevent horizontal transmission of *Salmonella* by minimizing its colonization in caeca. The positive impact of prebiotics is due to their potential in modulating the gut microbiota, making toll-like receptors that are important mediators of inflammation-related pathways in the gastrointestinal area [96].

#### 6.1.2. Probiotics

Probiotics are live and direct-fed microorganisms that provide health benefits to the host by modulating the immune system, if administered in adequate amounts [97]. Administration of probiotics to chicks soon after the hatch is thought to reduce infective pathogens due to ‘competitive exclusion’ [98]. The core concept of competitive exclusion is the competition of bacteria for space and nutrients and pathogenic microorganisms could be flushed out of the digestive tract via this mechanism. Several reports have documented the potential of probiotics to mitigate *Salmonella* infections [99]. Probiotics for poultry are mainly the various species of bacteria such as *Bifidobacterium*, *Lactobacillus*, and *Bacillus*.

#### 6.1.3. Phytobiotics

Phytobiotics are compounds of plant origin such as herbs, spices, and extracted oils that have shown promising effects on poultry production by increasing feed intake, stimulating the release of endogenous enzymes, and exerting antibacterial properties [100]. Moreover, they improve nutrient absorption by adjusting the fluidity and permeability of the cell membranes [101]. In the poultry industry, they are commonly obtained from alfalfa, bergamot, peppermint, black chili, cumin, clove, garlic, cinnamon, and oregano [28]. A drop in the *S. Enteritidis* population was observed in the liver/spleen and ceca of the broilers upon the inclusion of 5 ppm capsaicin [102].

#### 6.1.4. Essential Oils

Essential oils could be another practical feed additive to further combat *Salmonella*. Cinnamon, clove, oregano, and red thyme essential oils were shown to be efficacious for controlling the prevalence of the pathogen in poultry feed [103]. Utilization of clove, peppermint, litsea, lemongrass, and cinnamon essential oils were additionally investigated as an efficacious feed additive in the poultry industry [104]. Most recently, oregano, thyme, and grapefruit essential oils were studied as alternatives to antibiotics in poultry feed [105].

### 6.2. Non-Feeding Strategies

#### 6.2.1. Bacteriophages

Bacteriophages, also called phages, are known as predators of bacteria specific to the host and are considered as the alternative to antibiotics in animal therapy, prophylaxis, and minimizing bacterial population in animal-based food products [106]. Since they are typically host-specific, their therapeutic use is generally considered natural, non-toxic, and feasible, thus targeting the specific bacteria and protecting the rest of the microbiota [107]. Another advantage of phages over antibiotics is preventing allergies in the host as the immune system typically can tolerate phages more favorably [28]. Moreover, phages have the potential to combat antibiotic-resistant strains [108]. Some trials have shown the successful application of phages for mitigating foodborne pathogens such as *Salmonella* [109].

#### 6.2.2. Vaccines

Vaccines continue to be one of the most significant health-management strategies for boosting immunity in the poultry flock. Vaccination reduces shedding of *Salmonella* by poultry and could reduce vertical and horizontal transmission of the pathogen in the poultry chain [110]. In addition to these beneficial effects, vaccines are also the most efficacious and cost-effective tools to prevent diseases in birds [111]. The manufacturing of poultry vaccines is typically conducted using Typhimurium and/or Enteritidis serovars of *Salmonella* [112]. Different types of vaccines are available in the market, including live-attenuated, inactivated, and subunit vaccines, providing a range of options for disease prevention in poultry [113]. The production type, biosecurity status of the poultry premises, specific trend of the disease, level of maternal-derived antibodies, vaccine availability, administration methods, and vaccination cost are among the factors that play a crucial role in the application of this preventive measure. Typically, *Salmonella*-killed vaccines for poultry are preferred compared to the other vaccines due to the adverse effects that Salmonella-live vaccines could exert. The secondary mutations in live vaccines can cause reversion to virulence which could affect the overall health of flocks and thus should be carefully considered [114]. Both observational and randomized studies associated with the use of vaccines are very abundant in the literature and could be the subject of a review and systematic analysis in future studies.

#### 6.2.3. *In Ovo Strategies*

In ovo technology incorporates the administration of a small number of materials into the bird’s embryo, a new approach that delivers bioactive substances before hatching. Numerous studies have shown the benefits of in ovo strategies, at an early stage of development and involving feed additives, in avoiding metabolic disorders, compromised immunity, and pathogen load [115]. In addition to these, nutrients injected through the in ovo route enhance absorption, enzyme expression, and faster maturation of the digestive system and muscle tissues [116]. These beneficial effects directly and/or indirectly aid in the control of *Salmonella* infection. In a vaccination experiment, *Salmonella* flagellin was delivered to 18-day-old embryonated eggs in broilers. The study found elevation of inflammation-related chIL-6 and chIL-8 cytokine transcript levels 24 h after vaccination and elevated titers of FliC-specific antibodies 21 days after hatching [117]. In another experiment, increased resistance of chicks to *S. Typhimurium* was observed in broilers when the probiotic was injected into the air cells after 18 days of incubation [118].

### 6.3. Omics Tool for Identifying Antibiotic-Resistance Genes

The use of omics tools (studying the genome of organisms), such as whole genome sequencing (WGS) technology in agricultural production has led to advances in identifying genes associated with antibiotic-resistant bacteria. For instance, next-generation sequencing (NGS) technology has been applied to study over 30,000 genomes of *S. Enteritidis* from 98 countries over a span of 71 years to predict and understand the global dissemination of this pathogen [119]. Certain genes (such as TLR4), natural resistance-associated macrophage protein, and the QTL SAL1 in the genomic regions are central to controlling *Salmonella* infection in chickens [120]. 

### 6.4. Treatment of Salmonella in Poultry-Processing Facilities (Post-Harvest)

The journey of poultry from the farm to the consumer is a crucial aspect of the poultry industry. Any kind of contamination in poultry meat at this stage is correlated with negative impacts on public health. The duration for which these products can be stored before typical consumption ranges from 4 to 10 days, depending on the nature of processing after slaughter [121]. *Salmonella* is reported in higher proportion in fresh poultry products in comparison to other meat [122]. Thus, it is imperative that processors employ stringent measures to prevent *Salmonella* infection. In recent years, the reduction of pathogen contamination during processing has been achieved through post-chill decontamination tanks coupled with other preventive measures throughout the supply chain [123]. Antimicrobial strategies have been in implementation to minimize foodborne pathogens such as *Salmonella* and *Campylobacter* and to meet microbiological performance standards [124]. The potency of antimicrobials is influenced by the organic load present in the chilled water during immersion chilling [125]. Chemical applications such as chlorine, peracetic acid (PAA), and hydrogen peroxide are also effective in inhibiting microbial proliferation and prolonging the shelf-life of the product [126].

Chlorine has long been applied in processing facilities in the United States due to its cost-effectiveness and potential for eliminating a broad range of carcass microorganisms as well as microorganisms present in processing water and within processing plants [127]. However various studies have revealed the discouraging effects of chlorine including its negative impacts on meat quality, its sensitivity to pH changes, and its diminished effectiveness with time due to the presence of organic matter in the processing tank [128]. Peracetic acid is another organic compound commonly used to disinfect poultry equipment. However, attention should be paid to using peracetic acid in maximum concentrations, as it can corrode the equipment and bring health hazards to workers [129]. Sodium bisulfate may be a good substitute for peracetic acid against *Salmonella* in poultry facilities due to its less corrosive nature [130,131]. Other organic acids like lactic acid, citric acid, and acetic acid are also popular in meat-processing plants. While assessing the effectiveness of these organic acids on *S. Typhimurium* contaminated chicken meat, the reduction in CFU/g was observed to be 66%, 55%, and 51% for lactic acid (1% solution), acetic acid (1%), and citric acid (1%), respectively [132]. Additives and processing aids should be used after careful compliance with regulatory requirements to ensure efficacy of the treatments and safety of the workers and consumers.

### 6.5. Biosecurity at the Poultry Farm

Poultry production is compromised if there is no effective adoption of management and physical measures intended to mitigate the introduction and dissemination of infections or infestations in poultry premises and facilities [133]. The physical barriers used in robust biosecurity programs include fences, mesh wire, use of footbaths, and disinfection of farm equipment in and around poultry production facilities [134]. Biosecurity practices could be the most efficacious and inexpensive preventive measures, aimed at managing the risks posed by diseases to the economy, environment, and human health [135]. Overall, biosecurity measures are associated with isolation, traffic control, and sanitation practices [136]. Isolation addresses keeping the birds confined within a controlled environment. Traffic control looks at controlling the flow of traffic on a farm to mitigate cross-contamination and horizontal transmission of infectious diseases. The objective of sanitation is to clean and disinfect equipment and materials that enter or remain on the farms and it includes the personal hygiene of farm staff [137]. In addition to these biosecurity measures, sanitation of eggs, incubators, and the hatchery could substantially reduce the prevalence of microbial pathogens associated with poultry production. It is noteworthy that biosecurity measures not only minimize the risk of poultry-related infectious diseases but, as well, they are important from a regulatory perspective. Regulatory agencies such as United States Department of Agriculture’s Animal and Plant Health Inspection Services (USDA APHIS) or those regulations articulated for poultry farmers in the European Union are examples of reputable and efficacious policies and regulations if implemented properly. The Preventive Control for Animal Food regulation of the U.S. Food Safety Modernization Act could be another great resource for ensuring the safety of poultry products [138].

## 7. Conclusions

In a typical year, over one million American individuals and around 27 million people around the globe are estimated to contract non-typhoidal and typhoidal *Salmonella* infections, respectively. Considering that infectious diseases associated with Salmonellae and antibiotic resistance are expected to be augmented under the landscape of climate change, and considering that poultry-processing facilities and poultry products are one of the main reservoirs of *Salmonella* serovars, implementation of practical preventive measures is of great importance for reducing the public health burden of this pathogen. Feeding-based strategies (including use of prebiotics, probiotics, phytobiotics, and/or essential oils), non-feeding-based strategies (such as application of bacteriophages, vaccinations, and in ovo interventions), omics tools and surveillance for identifying antibiotic-resistance genes, post-harvest application of antimicrobials, and enhanced biosecurity at the poultry facilities are practical interventions that could reduce the public health burden of salmonellosis and antibiotic resistance associated with poultry products.
